# Probable Acquired Autosplenectomy in Systemic Lupus Erythematosus and Sjögren’s Overlap Syndrome: A Case Report With Serial Imaging Evidence

**DOI:** 10.7759/cureus.109958

**Published:** 2026-05-31

**Authors:** Khalid Shahzad, Zainab Asghar

**Affiliations:** 1 Internal Medicine, King Edward Medical University, Lahore, PAK; 2 Internal Medicine, Queen Elizabeth Hospital, Gateshead, GBR

**Keywords:** autosplenectomy, hyposplenism, sjögren’s syndrome, systemic autoimmune disease, systemic lupus erythematosus

## Abstract

Autosplenectomy, defined as the spontaneous loss of splenic tissue in the absence of trauma or surgery, is most commonly associated with sickle cell disease and is rarely described in patients with autoimmune conditions. We present a case of a 54-year-old woman with systemic lupus erythematosus (SLE) and Sjögren’s overlap syndrome who was incidentally found to have a complete absence of the spleen on computed tomography (CT), despite prior imaging demonstrating a normal splenic anatomy. The patient presented with polyarthralgia, fatigue, and systemic inflammation. Laboratory investigations revealed elevated anti-dsDNA titers, hypocomplementemia, and a positive extractable nuclear antigen (ENA) profile. Imaging confirmed splenic absence, and extensive evaluation excluded infectious, malignant, infiltrative, and hematological causes of splenic agenesis. Serial imaging findings, combined with the presence of an active autoimmune disease, supported the diagnosis of a probable autoimmune-mediated autosplenectomy. This case highlights a rare but clinically significant manifestation of systemic autoimmune diseases. Recognition of asplenia is critical because of the associated risk of overwhelming infection, necessitating vaccination and the implementation of prophylactic strategies. Clinicians should consider splenic dysfunction in patients with autoimmune diseases, particularly when unexplained infections or atypical imaging findings are present.

## Introduction

Autosplenectomy refers to the spontaneous atrophy and functional loss of the spleen in the absence of prior surgical removal or traumatic injury. It is most commonly described in sickle cell disease, where repeated vaso-occlusive episodes result in progressive splenic infarction and fibrosis [1]. In contrast, autosplenectomy in autoimmune conditions, such as systemic lupus erythematosus (SLE) and Sjögren’s syndrome, is exceedingly rare. Functional hyposplenism has been reported in SLE, with studies demonstrating impaired splenic function and increased susceptibility to infection; however, radiologically confirmed, complete anatomical absence of the spleen remains exceptionally uncommon [2-5].

The pathophysiological mechanisms underlying splenic atrophy in autoimmune diseases are not fully understood but are thought to involve immune complex-mediated vasculitis, microvascular thrombosis, and chronic inflammatory damage, leading to progressive fibrosis and infarction [2,6,7]. Similarly, splenic dysfunction has been associated with Sjögren’s syndrome, with case reports highlighting the risk of severe infection in the context of unrecognized hyposplenism [8]. Broader reviews of hyposplenism further emphasize that autoimmune and inflammatory conditions can contribute to progressive splenic dysfunction through immune-mediated vascular injury [9,10].

We present a unique case of autoimmune-mediated autosplenectomy in a patient with SLE/Sjögren’s overlap syndrome, supported by serial imaging demonstrating progression from normal splenic anatomy to the absence of splenic tissue. This case highlights an under-recognized complication of systemic autoimmunity with significant diagnostic and clinical implications [2-5,8-10].

## Case presentation

A 54-year-old woman with a known history of Sjögren’s syndrome, who had been off hydroxychloroquine therapy for approximately two years, presented with a two-week history of worsening polyarthralgia, generalized fatigue, and systemic malaise. There was no history of recent trauma, surgery, or hematological disorders.

On clinical examination, the patient had active synovitis involving multiple joints and bilateral lower limb edema. She appeared systemically unwell, with features suggestive of an inflammatory process. Initial laboratory investigations revealed elevated inflammatory markers, including a C-reactive protein (CRP) level of 66.9 mg/L. Hematological parameters revealed anemia (hemoglobin level: 92 g/L) and mild leukocytosis. Renal function was impaired, and the creatinine levels were significantly elevated (Table 1).

**Table 1:** Baseline hematological and biochemical investigations on admission. CRP, C-reactive protein; ALT, alanine aminotransferase; AST, aspartate aminotransferase

Test	Result	Reference Range
White cell count	12.5 ×10⁹/L	4.0-11.0 ×10⁹/L
Neutrophils	8.91 ×10⁹/L	2.0-7.5 ×10⁹/L
Hemoglobin	92 g/L	115-165 g/L
Platelets	358 ×10⁹/L	150-400 ×10⁹/L
CRP	66.9 mg/L	0-5 mg/L
Urea	11.4 mmol/L	2.5-7.8 mmol/L
Creatinine	396 µmol/L	60-120 µmol/L
Sodium	133 mmol/L	133-146 mmol/L
Potassium	4.4 mmol/L	3.5-5.3 mmol/L
ALT	55 U/L	0-40 U/L
AST	44 U/L	0-40 U/L
Albumin	28 g/L	35-50 g/L

Immunological workup showed hypocomplementemia (reduced complement component 3 (C3) and complement component 4 (C4) levels) and markedly elevated anti-double-stranded DNA (anti-dsDNA) titers ranging from 134 IU/mL to 176 IU/mL. The extractable nuclear antigen (ENA) panel was positive for anti-Ro and anti-La antibodies, consistent with active SLE in the context of Sjögren’s syndrome overlap (Table 2).

**Table 2:** Autoimmune and immunological profile. ANA, antinuclear antibody; ENA, extractable nuclear antigen; anti-dsDNA, anti-double-stranded DNA; SSA, Sjögren syndrome-related antigen A (Ro); SSB, Sjögren syndrome-related antigen B (La); RNP, ribonucleoprotein; Sm, Smith antigen; C3, complement component 3; C4, complement component 4

Test	Result	Reference Range
ANA	Positive	Negative
ENA	Positive	Negative
Anti-dsDNA	134 IU/mL	0-99 IU/mL
Anti-Ro (SSA)	Positive	Negative
Anti-La (SSB)	Positive	Negative
Anti-RNP/Sm	Positive	Negative
C3	0.40 g/L	0.90-1.80 g/L
C4	0.03 g/L	0.10-0.40 g/L
Beta-2 microglobulin	7.5 mg/L	0.0-2.2 mg/L

The patient was started on intravenous methylprednisolone for a presumed lupus flare. However, the clinical response was suboptimal. Blood cultures were positive for *Staphylococcus aureus*, and the patient was started on intravenous administration of flucloxacillin. A new systolic murmur prompted further evaluation using transesophageal echocardiography, which excluded the presence of infective endocarditis. 

**Figure 1:**
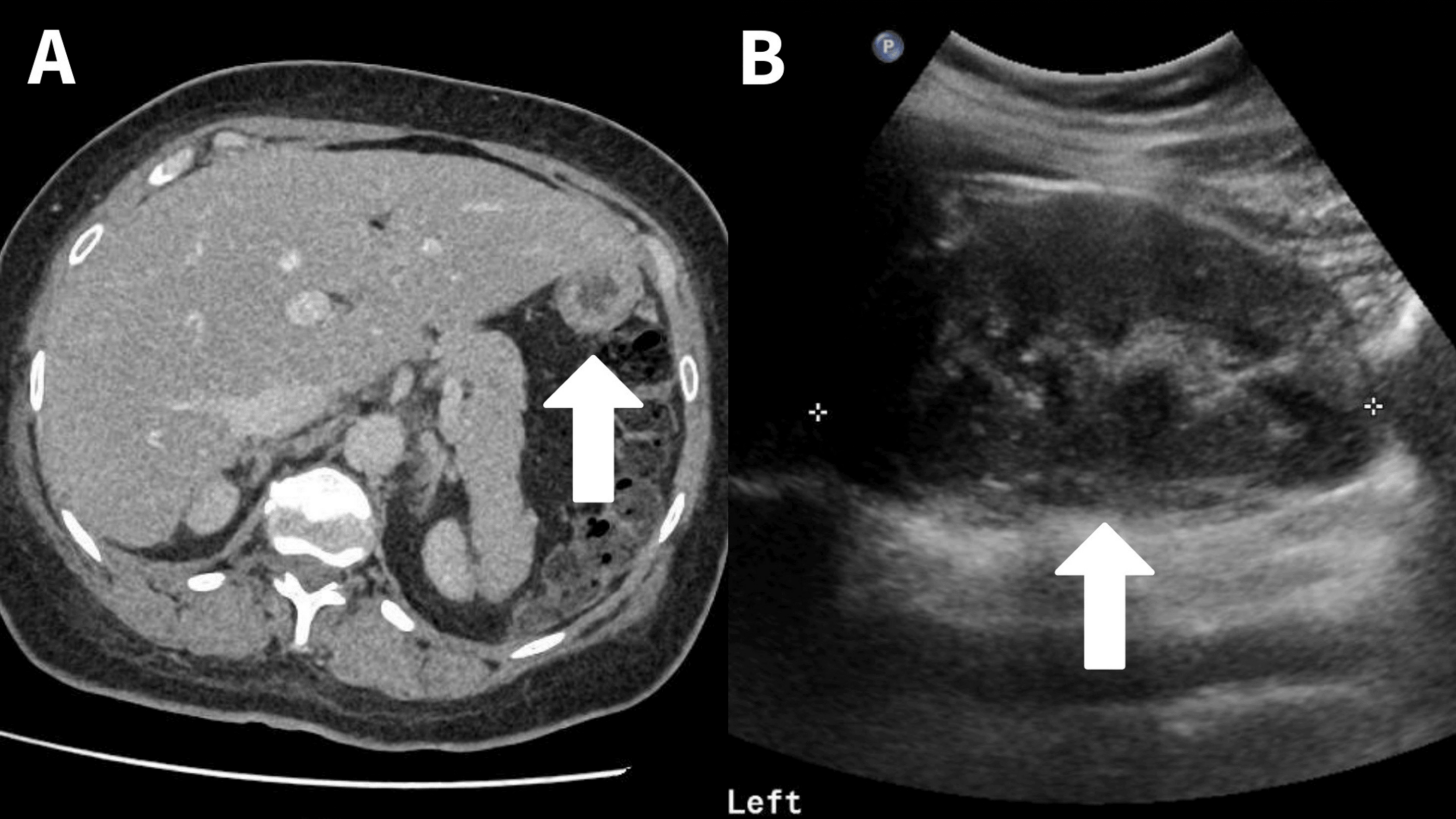
Evolution of splenic findings on imaging. (A) CT abdomen (2025) showing absence of splenic tissue. The arrow indicates the expected splenic bed/left upper quadrant, highlighting the absence of splenic tissue (some redundant tissue visible). (B) Ultrasound image (2011) demonstrating a normal spleen. The arrow indicates the normal spleen, confirming that splenic anatomy was previously preserved. CT, computed tomography

Computed tomography (CT) scan of the chest, abdomen, and pelvis (CT TAP) performed during this admission revealed the absence of splenic tissue in the left upper quadrant (Figure 1A). A review of prior imaging demonstrated a normal spleen on ultrasound performed in 2011 (Figure 1B), confirming that splenic loss was acquired.

Further imaging findings revealed mesenteric panniculitis and multiple enlarged lymph nodes, raising the suspicion of an underlying lymphoproliferative disorder. However, subsequent lymph node biopsy revealed reactive changes without evidence of malignancy.

**Figure 2:**
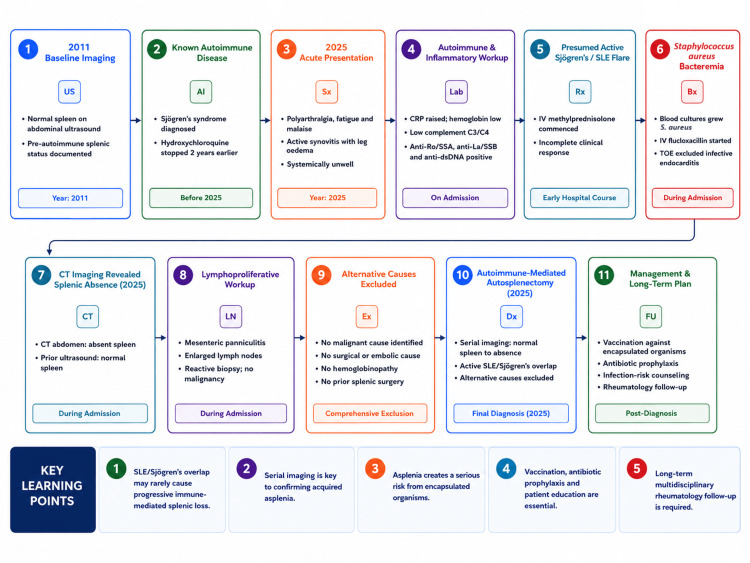
Clinical progression and diagnostic journey of probable autoimmune-mediated autosplenectomy in SLE/Sjögren’s overlap syndrome. Timeline illustrating the evolution from previously documented normal splenic anatomy (2011 ultrasound) to complete absence of splenic tissue on 2025 CT imaging, alongside key clinical features, autoimmune workup, exclusion of alternative etiologies, and subsequent management with asplenia prophylaxis. SLE, systemic lupus erythematosus; SSA, Sjögren syndrome-related antigen A (Ro); SSB, Sjögren syndrome-related antigen B (La); TOE, transesophageal echocardiography; CRP, C-reactive protein; C3, complement component 3; C4, complement component 4; anti-dsDNA, anti-double-stranded DNA; IV, intravenous; US, ultrasound; AI, autoimmune disease; Sx, symptoms; Lab, laboratory workup; Rx, treatment; Bx, blood culture/bacteremia; CT, computed tomography; LN, lymph node; Ex, exclusion of alternative causes; Dx, diagnosis; FU, follow-up The figure was created using Canva (Canva Pty Ltd., Sydney, Australia).

A comprehensive evaluation was performed to identify the potential causes of splenic atrophy in this patient. Infectious etiologies, including tuberculosis, were excluded based on negative cultures and the absence of radiological or clinical features suggestive of a granulomatous disease. Infiltrative conditions such as sarcoidosis and amyloidosis were considered unlikely because of the absence of characteristic systemic involvement. Hematological causes, including lymphoma and hemoglobinopathies, were excluded based on laboratory findings and histopathology. The patient had no history of trauma or splenic surgery (Figure 2). 

Given the absence of alternative etiologies, the presence of an active autoimmune disease, and documented progression from normal splenic anatomy to complete absence on serial imaging, a diagnosis of autoimmune-mediated autosplenectomy in SLE/Sjögren’s overlap syndrome was made. 

In view of the confirmed asplenic state, the patient was initiated on appropriate post-splenectomy prophylactic measures, including vaccination against encapsulated organisms and counseling regarding infection risk.

## Discussion

Autoimmune-mediated autosplenectomy is an exceptionally rare clinical condition. While functional hyposplenism has been described in SLE, an anatomical absence of the spleen has seldom been reported [2-5]. The pathophysiology of splenic atrophy in autoimmune diseases is not fully understood but is believed to involve a combination of immune complex deposition, small vessel vasculitis, and microvascular thrombosis, leading to repeated splenic infarction and progressive fibrosis [2,6,7]. Early studies by Dillon et al. provided histopathological and imaging evidence supporting immune-mediated vascular injury as a key mechanism of splenic atrophy in patients with SLE [2,3].

Recent literature has reinforced that hyposplenism in autoimmune diseases may represent a spectrum ranging from functional impairment to complete anatomical loss [9,10]. In addition, studies have demonstrated that patients with SLE may develop clinically significant splenic dysfunction, predisposing them to an increased risk of infection, even in the absence of overt anatomical changes [4,5]. Cases associated with Sjögren’s syndrome further support the role of chronic inflammation and immune dysregulation in this condition. Santos et al. described a patient with Sjögren’s syndrome and acquired splenic atrophy complicated by septic shock, underscoring the potential consequences of unrecognized hyposplenism [8].

In contrast to previously reported cases, which primarily describe functional hyposplenism or partial atrophy, our patient demonstrated a radiologically confirmed absence of the spleen, supported by prior imaging showing normal splenic anatomy [2-5,8]. This provides strong evidence for a progressive autoimmune-mediated process rather than congenital absence or incidental findings. To our knowledge, such cases demonstrating documented progression from a normal spleen to complete autosplenectomy in patients with autoimmune overlap syndromes are exceedingly rare [2-5,8-10].

The differential diagnosis of splenic atrophy is broad and includes infiltrative diseases such as sarcoidosis and amyloidosis, granulomatous infections such as tuberculosis, hematological malignancies including lymphoma, and hemoglobinopathies such as sickle cell disease [6,7,11]. These conditions were systematically excluded in our patient through a combination of imaging, laboratory testing, and histopathological examinations.

The clinical implications of autosplenectomy are therefore significant. The spleen plays a crucial role in immune defense, particularly in clearing encapsulated organisms from the body. Patients with asplenia or hyposplenism are at an increased risk of overwhelming post-splenectomy infection (OPSI), which carries substantial morbidity and mortality risks [7,12]. Therefore, early recognition is essential to ensure appropriate vaccination against pneumococcus, meningococcus, and *Haemophilus influenzae*, as well as consideration of antibiotic prophylaxis [12-14].

Current guidelines emphasize the importance of preventive strategies, including immunization, patient education, and long-term follow-up, in individuals with absent or dysfunctional spleens [12-14]. This case highlights the importance of reviewing prior imaging in patients with autoimmune diseases and considering splenic dysfunction when evaluating unexplained infections or atypical imaging findings. This underscores the need for increased awareness of this rare but clinically important complication of autoimmune overlap syndromes [2-5,8-10].

## Conclusions

This case highlights a rare but clinically significant phenomenon of autoimmune-mediated autosplenectomy in a patient with overlap of SLE and Sjögren’s syndrome. Serial imaging confirmed splenic absence without prior surgical or traumatic causes. This underscores the need for clinicians to monitor splenic function in patients with autoimmune diseases, particularly those at risk of infection, sepsis, or immunological complications. Timely recognition allows for appropriate vaccination, prophylaxis, and patient counseling to mitigate the risks associated with asplenia.
